# Uniformity Correction of CMOS Image Sensor Modules for Machine Vision Cameras

**DOI:** 10.3390/s22249733

**Published:** 2022-12-12

**Authors:** Gabor Szedo Becker, Róbert Lovas

**Affiliations:** 1 Doctoral School of Applied Informatics and Applied Mathematics, Óbuda University, Bécsi út 96/B, 1034 Budapest, Hungary; 2 Institute for Computer Science and Control (SZTAKI), Eötvös Loránd Research Network (ELKH), Kende u. 13-17, 1111 Budapest, Hungary

**Keywords:** CMOS, image sensor, ISP, FPGA, ASIC, NUC, FFC, FPN, DSNU, PRNU

## Abstract

Flat-field correction (FFC) is commonly used in image signal processing (ISP) to improve the uniformity of image sensor pixels. Image sensor nonuniformity and lens system characteristics have been known to be temperature-dependent. Some machine vision applications, such as visual odometry and single-pixel airborne object tracking, are extremely sensitive to pixel-to-pixel sensitivity variations. Numerous cameras, especially in the fields of infrared imaging and staring cameras, use multiple calibration images to correct for nonuniformities. This paper characterizes the temperature and analog gain dependence of the dark signal nonuniformity (DSNU) and photoresponse nonuniformity (PRNU) of two contemporary global shutter CMOS image sensors for machine vision applications. An optimized hardware architecture is proposed to compensate for nonuniformities, with optional parametric lens shading correction (LSC). Three different performance configurations are outlined for different application areas, costs, and power requirements. For most commercial applications, the correction of LSC suffices. For both DSNU and PRNU, compensation with one or multiple calibration images, captured at different gain and temperature settings are considered. For more demanding applications, the effectiveness, external memory bandwidth, power consumption, implementation, and calibration complexity, as well as the camera manufacturability of different nonuniformity correction approaches were compared.

## 1. Introduction

Digital images captured by image sensors are contaminated with noise, which deteriorate performance and reduce sensitivity. Image noise can be characterized as temporal or lateral fixed-pattern noise (FPN). Temporal noise changes from frame to frame, while FPN is mostly constant, but may depend on temperature or sensor configuration.

### 1.1. A Linear Model of Spatial Nonuniformity

The mathematical framework for analysis was introduced by Mooney [1] and later simplified by Perry [2] for the linear model of FPN for infrared focal plane arrays. Though Schulz [3] expanded the nonuniformity correction (NUC) to multipoint analysis to account for the nonlinearities of IR FPNs, the linearity of the CMOS image sensor photoresponse allows a generalization of Mooney’s framework for CMOS imagers. The luminous flux received by a small surface element with *A*, exposed to irradiance *L*, with incident angle Θ is

(1) P=LAΘ,

Without considering the effect of temporal noise sources, such as electronic, thermal, and shot noise, the number of electrons present on the cathode of a reverse-biased photodiode illuminated by a narrow-band light source can be modeled by:

(2) $$N=T_{int}\left(\eta P+D\right)+Q_{R},$$

where $T_{int}$ is the integration time, η is the quantum efficiency, assumed constant for the narrow spectrum of the illuminator, *D* is the dark current, and $Q_{R}$ is the residual charge present on the cathode after reset. CMOS image sensors use correlated double sampling (CDS) which effectively cancels out the $Q_{R}$ term [3]. For a pixel with area *A*, at position x,y in the pixel array, illuminated via a lens by a wideband illuminator, the number of electrons collected by the pixel can be expressed as

(3) $$N_{x,y}=T_{int}\left[\tau_{x,y}\int_{\lambda_{1}}^{\lambda_{2}}L_{x,y}\left(\lambda \right)\eta_{x,y}\left(\lambda \right)\;d\lambda A\Omega_{x,y}\right]+D_{x,y},$$

where $L_{x,y}$, $\eta_{x,y}$, and $D_{x,y}$ are the spectral radiance density, the quantum efficiency, and the residual dark offset specific for pixel x,y, respectively, while

(4) $$\Omega_{x,y}=\left[\frac{\pi \cos^{4}\Theta_{x,y}}{4F^{2}+1}\right],$$

is the projected solid angle subtended by the exit pupil of the optical system, as viewed from the sensor pixel x,y, where $\Theta_{x,y}$ is the off-axis angle of the pixel and *F* is the F-number of the lens. The transmittance of the optical system is assumed to be homogeneous in Mooney’s model; however, for many CMOS cameras, optical efficiency tends to drop towards the corner of the image due to the chief ray angle (CRA) mismatch between the last lens element and the microlens array focusing light onto the photodiodes. Hence, the optical efficacy, $\tau_{x,y}$, is dependent on pixel position and is an important source of fixed-pattern nonuniformity. With the introduction of a response coefficient,

(5) $$R_{x,y}=A\tau_{x,y}\left[\frac{\pi \cos^{4}\Theta_{x,y}}{4F^{2}+1}\right],$$

(3) can be simplified to

(6) $$N_{x,y}=T_{int}\left[R_{x,y}\int_{\lambda_{1}}^{\lambda_{2}}L_{x,y}\left(\lambda \right)\eta_{x,y}\left(\lambda \right)\;d\lambda \right]+D_{x,y},$$

To analyze the impact of pixel-to-pixel variation of parameters, the parameters can be expressed as:

(7) $$D_{x,y}=\langle D\rangle +d_{x,y},$$

(8) $$R_{x,y}=\langle R\rangle +r_{x,y},$$

(9) $$\eta_{x,y}\left(\lambda \right)=\langle \eta \left(\lambda \right)\rangle +\kappa_{x,y}\left(\lambda \right),$$

where the bracketed variables denote the mean value of the corresponding parameter across the entire image, and the additive quantities capture the pixel-to-pixel variations. Namely, $d_{x,y},r_{x,y},$ and $\kappa_{x,y}\left(\lambda \right)$ are the pixel-to-pixel variation in dark current, response coefficient, and quantum efficiency, respectively. When capturing an image with zero illumination, $L_{x,y}\left(\lambda \right)=0$, referred to as the dark image, (6) yields

(10) $$N_{x,y}=D_{x,y}=\langle D\rangle +d_{x,y},$$

the time-invariant pixel-to-pixel nonuniformity $d_{x,y}$, referred to as dark signal nonuniformity (DSNU), with variance $\sigma_{d}$.

When looking at a uniform gray field, often referred to as a flat field (FF), with $L_{x,y}\left(\lambda \right)=L$, (6) yields

(11) $$N_{x,y}=T_{int}\left[R_{x,y}L\int_{\lambda_{1}}^{\lambda_{2}}\left(\lambda \right)\eta_{x,y}\left(\lambda \right)\;d\lambda \right]+D_{x,y},$$

By substituting (7)–(9) into (11),

(12) $$N_{x,y}=T_{int}\left[\left(\langle R\rangle +r_{x,y}\right)L\int_{\lambda_{1}}^{\lambda_{2}}\langle \eta \left(\lambda \right)\rangle +\kappa_{x,y}\left(\lambda \right)d\lambda \right]+\langle D\rangle +d_{x,y},$$

which in turn can be separated to a spatially uniform part, the perfectly reproduced, constant, flat field:

(13) $$N\left(L\right)=T_{int}\left[\langle R\rangle L\int_{\lambda_{1}}^{\lambda_{2}}\langle \eta \left(\lambda \right)\rangle \left(\lambda \right)d\lambda \right]+\langle D\rangle ,$$

and another term constituting the fixed-pattern noise:

(14) $$N_{x,y}=T_{int}\left[Lr_{x,y}\int_{\lambda_{1}}^{\lambda_{2}}\langle \eta \left(\lambda \right)\rangle +\kappa_{x,y}\left(\lambda \right)d\lambda \right]+d_{x,y},$$

the first term of which is referred to as the photoresponse nonuniformity (PRNU). The variance of the PRNU, following the derivation in [2], assuming $\kappa_{x,y}\left(\lambda \right)$, $d_{x,y}$, and $r_{x,y}$ are statistically independent, can be expressed as:

(15) $$\sigma_{n}^{2}=\sigma_{D}^{2}+\frac{\sigma_{R}^{2}}{{\langle R\rangle }^{2}}\left(N\left(L\right)-\langle D\rangle \right)+\langle R\rangle L\int_{\lambda_{1}}^{\lambda_{2}}\kappa_{x,y}\left(\lambda \right)d\lambda ,$$

### 1.2. FPN Noise Reduction in CMOS Sensors

**Figure 1:**
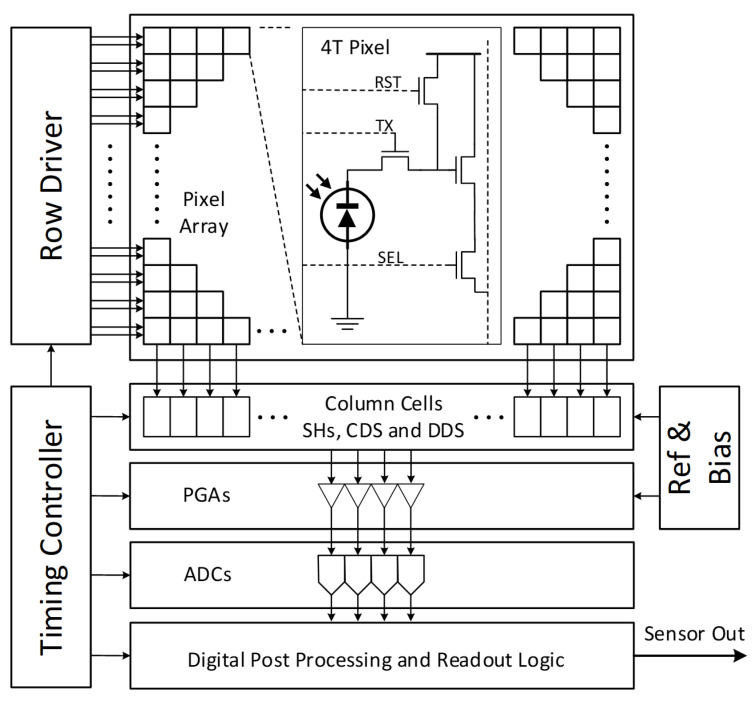
Typical CMOS image sensor block diagram.

Figure 1 shows the typical structure of a CMOS image sensor.

**Figure 2:**
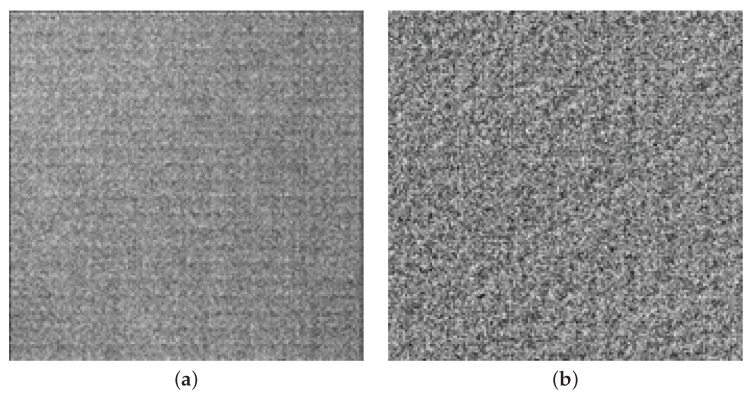
FPN of the IMX265LLR-C at 60 °C and 12 dB of analog gain: (**a**) no zoom and (**b**) 8× zoom.

A matrix of pixels can collect electrons generated by the photoelectric effect. A row of pinned photodiodes can be reset, exposed, and read out by corresponding row reset, row transfer, and select drivers. Multiple pixels of a row can be read out simultaneously via a set of programmable gain amplifiers (PGAs) and analog to digital converters (ADCs). Modern CMOS sensors use correlated double sampling (CDS, Nakamura [4]) or correlated multiple sampling (CMS) (Min-Woong [5]). Analog or digital hardware solutions eliminate dark charge $Q_{R}$, by sampling and holding the output of a pixel after reset, then sampling the same output during readout. The column amplifier outputs the difference between the two samples, which effectively removes any common mode bias, such as reset noise. Differential delta sampling (DDS) aims to remove fixed-pattern noise introduced by small differences between the sample-and-hold (SH) capacitors, and biases of the programmable gain amplifiers using a crowbar operation (Kim [6]). Before CDS and DDS, column-wise readout via PGAs and ADCs and row-wise addressing gave the DSNU and PRNU a characteristic striated, row–column-oriented structure shown on the left-side image of Figure 2. The right-side image shows a magnified, contrast-enhanced sample enhanced for visibility.

Another often-used technique to remove DSNU is to use the optically masked pixels surrounding the active pixel area. These pixels are affected by the same conditions (temperature, electronic noise, analog gain) as the active pixels, and their values could be digitally subtracted from the corresponding rows and columns, further reducing systemic, row–column-structured noise

### 1.3. Temperature Dependence

The temperature dependence of dark current in silicon photodiodes (Takayanagi [7]) can be expressed as:

(16) $$I_{D}\left(T\right)=A_{dgen}T^{1.5}e^{-\frac{-E_{g}}{2kT}}+B_{diff}T^{3}e^{-\frac{-E_{g}}{kT}},$$

where $E_{g}$ is the activation energy, *k* is the Boltzmann constant, and $A_{dgen}$ and $B_{diff}$ are technology dependent coefficients. The shape of the aggregate temperature dependence function has two exponential regions, one dominated by the diffusion current and one by the spontaneous generation current.

## 2. Motivation

This section provides an overview of the application areas benefiting from an improved uniformity of image sensors.

### 2.1. Image and Video Quality Improvements for Enhanced Viewer Experience

While this analysis focuses on large pixel, high-quality, low-noise, global shutter sensors, many consumer products use small, low-cost CMOS sensors. Smaller pixels often have a reduced full well capacity and in turn a smaller dynamic range. Video recorded from sensors contains temporal and fixed-pattern noise superimposed on the signal. The human visual system easily disregards the temporal, Gaussian noise, but discerns patterned FPN deeply buried in temporal noise. FPN is particularly disturbing when it is superimposed on human faces in video conferencing. As viewers track facial features, movements of the face relative to the image sensor causes an apparent shift of FPN artifacts over the subject, which most viewers notice and may find objectionable.

### 2.2. Astronomy and LIDAR

On the other end of the sensor price/quality spectrum, large, stabilized focal plane arrays are used to image celestial objects. High-end staring cameras typically track their targets and use extended exposure intervals or collect many exposures to form output images then register the image stacks. Image stacking may amplify DSNU if motion between constituent frames is negligible relative to the spatial frequencies of the DSNU. Scheimpflug Lidars using CMOS sensors (Mei [8]) also depend on FFC to improve SNR.

### 2.3. Visual Odometry

In machine vision camera applications, the consumer of video streams are algorithms, which may be less effective at canceling noise than the human visual system. Especially for high-frame-rate imaging tasks with short integration time, the relatively low signal-to-noise ratio calls for digital postprocessing of the images to reduce FPN. A prime example of this use case is disparity mapping for visual odometry. In this case, two image sensors, with different FPN profiles are looking at the scene. The processing algorithm infers depth or the z-axis distance from the sensor pair from the difference or disparity between the images. Disparity mapping attempts to find a correspondence between an image pair, and often sub-pixel differences in x–y plane disparities are amplified when estimating the z-axis depth.

**Figure 3:**
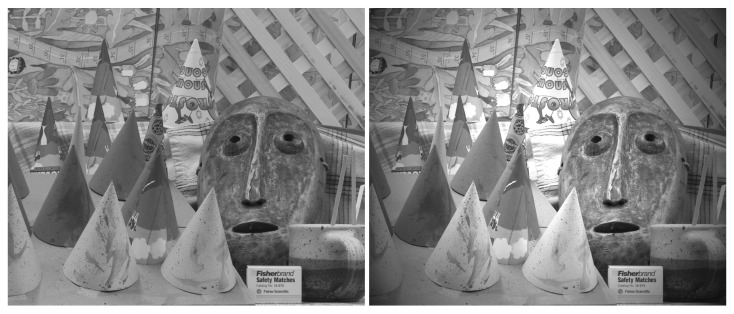
Cone dataset original (**left**) and with FPN (**right**).

**Figure 4:**
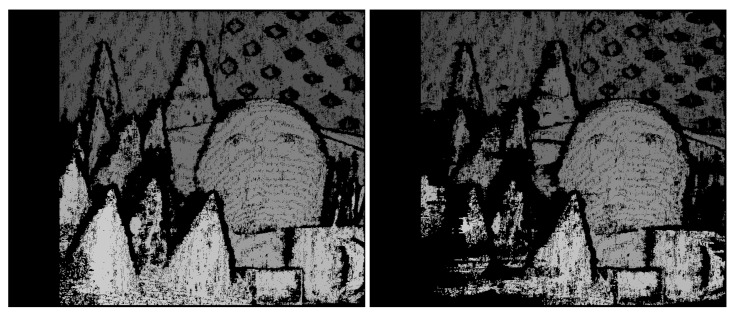
Teddy dataset original (**left**) and with FPN (**right**).

The analysis of stereo image pairs collected using a low-cost sensor (EV76C661 [9]) showed that matching density improved from 10.2% to 13.1% with FFC enabled, a 28% improvement. Expected improvements in matching performance can be simulated without the actual image sensor in hand. If detailed FPN information is available, e.g., by performing the EMVA1288 [10] analysis of a sensor, then synthetic image pairs with ground-truth depth information, such as the Middlebury dataset (Scharstein [11]), can be analyzed with and without FPN (Figure 3). Similar to lab results with actual image sensors, matching densities without lens shading, DSNU, and PRNU were 28% better for both the cone and teddy datasets (Figure 4).

### 2.4. Forensics

To extract DSNU and PRNU from live video or image frames using nonuniform illumination, different algorithmic solutions have been proposed, based on regularization (Li, [12]), or convolutional neural networks (Guan, [13]). Using a large number of frames, especially with large image areas with frequency content sufficiently different from the spectra of the DSNU and PRNU, such as images of the sky, allows the recovery of the DSNU and PRNU. The same techniques can be used to identify the source of a video, by matching FPN as a watermark, embedded in the sources. One application of FFC is to promote data privacy by reducing FPN to a level below the capabilities of forensic algorithms (Karaküçük [14]).

## 3. Materials and Methods

### 3.1. Image Capture Parameters

At least two exposures, one with no illumination, and one with a flat uniform illumination are necessary to capture the sensor-specific correction images. For low-noise, CMOS, visible light sensors with improved image sensor circuitry (7 transistor pixels, CDS, DDS), thermal, electrical, and shot noise can be several orders of magnitude larger than FPN. In order to cancel temporal noise and to measure DSNU and PRNU, thousands of images need to be captured and averaged. To analyze the temperature and analog gain level dependency of the DSNU and PRNU, the capture sequence was performed in a temperature-controlled environment, with different gain settings. Specifically, images were captured

- For two 2nd generation, global shutter, monochrome, Sony Pregius machine vision sensor candidates, the IMX265LLR-C and the IMX273LLR-C;
- Across the entire analog gain range supported by the two sensor candidates, at 0.0, 6.0, 12.0, 18.0 and 24.0 dB;
- For the above datasets, for both sensors, for 5 gain settings, via the temperature range supported by the sensors, at 0.0, 15.0, 30.0, 45.0, and 60 Celsius degrees.

The dataset was collected with the Sony IMX265LLR-C, with the lens and lens housing removed. A smaller dataset, with two temperatures (10 °C and 50 °C) and two analog gain settings (2.0 dB and 24.0 dB), was collected for both the IMX265 and IMX273, with two different lens assemblies attached to the sensor PCBA. For each sensor, gain, and temperature setting, the mean of the collected image stack:

(17) $$\mu_{x,y}\left(\bar{p}\right)=\frac{1}{N}\sum_{1}^{N}F_{x.y}\left(\bar{p}\right),$$

and the standard deviation of the stack was computed as

(18) $$\sigma_{x,y}\left(\bar{p}\right)=\frac{1}{N-1}\sqrt{\sum_{1}^{N}F_{x.y}^{2}\left(\bar{p}\right)-N\mu_{x,y}^{2}\left(\bar{p}\right)},$$

where $\bar{p}=\left[T,\alpha ,s_{ID},t\right]$ is a parameter vector of the capture temperature (*T*), analog gain (α), sensor ID ($s_{ID}$), and exposure time (*t*). $F_{x,y}^{2}\left(\bar{p}\right)$ and $\mu_{x,y}^{2}$ designate the element-wise squaring of pixel values for FF frame $F_{x,y}\left(\bar{p}\right)$ and image stack mean frame $\mu_{x,y}\left(\bar{p}\right)$. To reduce temporal noise below 1 LSB, *N* = 4000 images had to be averaged for each parameter combination.

### 3.2. Instrumentation

In order to precisely control the temperature of the sensor, the sensor module was mounted on a 24 V 30 W flexible polyimide heating film, connected to a USB controllable power supply (Keysight E3634A). To control sensor temperature, a software-defined PID controller was employed, using the built-in thermometer function of the sensor.

**Figure 5:**
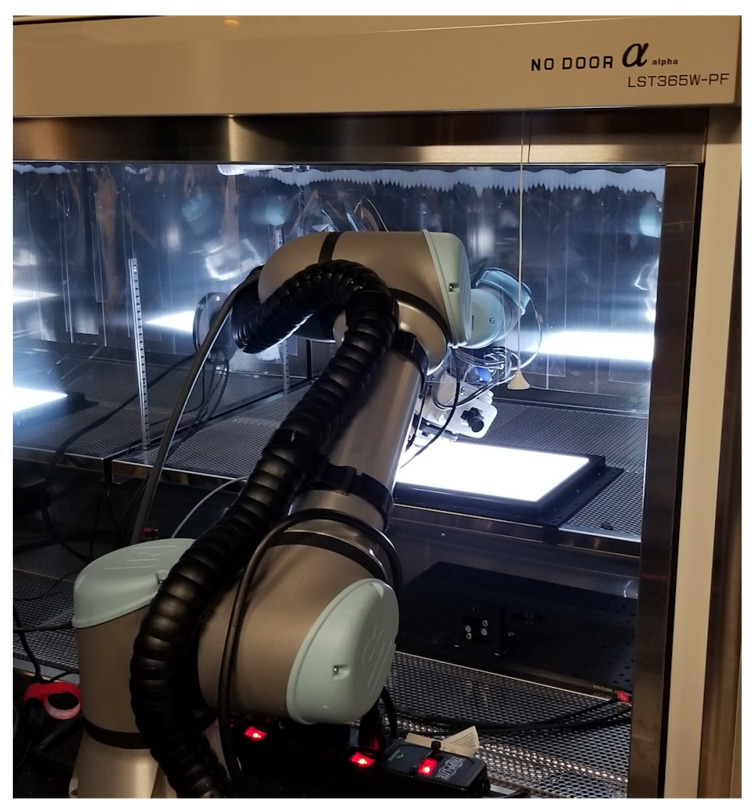
Sensor assembly on robot arm in temperature chamber.

Data collection took place in a temperature chamber (No Door α LST365W-PF), which could cool the sensor down to 0 °C. As a FF light source, an LED panel, Imaging Tech Innovation model ITLB-ST-V1-100K, was used. The thermoelectric heating/cooling plate and the sensor module were housed in a custom designed, 3D-printed enclosure, which attached the sensor assembly to a flexible, collaborative robot arm (Universal Robot UR5e), programmed to move the sensor assembly on a closed, circular trajectory (Figure 5).

The introduction of motion while the image stack was recorded was necessary to blur any nonuniformity attributable to the light source. This was essential for the dataset with properly focused lens assemblies attached to the sensor. While many other results published (Burggraaff [15]) were based on measurements with integrating spheres, experiments with the lens assembly attached and properly focused revealed both the low-frequency (shading), and the high-frequency (contamination) characteristics of our integrating sphere. Wang [16] also documented and addressed this issue. The specified uniformity of laboratory integrating spheres, typically in the 40 dB range [17], is insufficient for testing 12-bit sensors.

**Figure 6:**
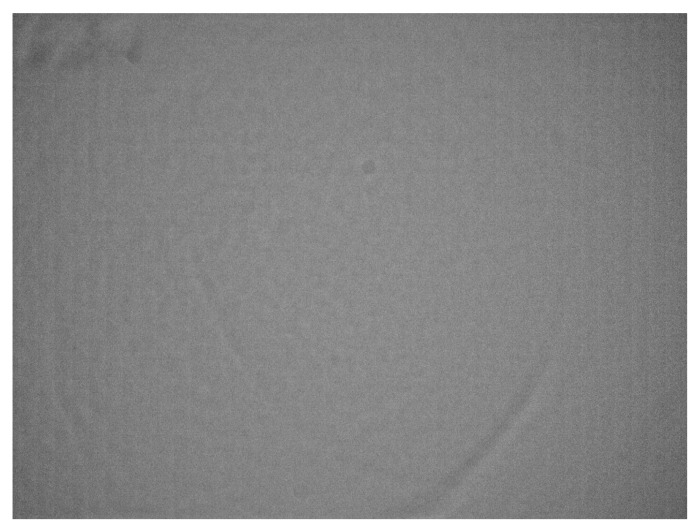
Static integrating sphere’s image artifacts.

With lens shading corrected and the image normalized for viewing, the high-frequency content of the integrating sphere nonuniformity was revealed (Figure 6). If the image sensor was repositioned in the viewport, the PRNU component remained fixed, but smudges and other artifacts were shifted.

## 4. Related Work

As mentioned in the introduction, the linear model framework for FFC was introduced by Mooney [1] and Perry [2]. Generalized linear correction architectures similar to (19) were proposed in the seminal works of Seibert [18] and Snyder [19].

Most FFC approaches are based on static dark-frame and flat-field calibration images, but dynamic methods, using scene-based FFC have also been proposed [20]. While scene-based methods require no calibration prior to use, significant back-end image processing is necessary to discern non-striated nonuniformities to be suppressed from the dynamic scene content. Convolutional neural networks (CNN) can also be used to identify and suppress FPN [13]; however, this method also requires dedicated HW resources or CNN accelerators made available for the embedded ISP platform. With either method, ISP architects are trading static calibration time, invested during manufacturing, with initialization time spent after each power-on, while the system dynamically calibrates to discern sensor-specific FPN. It is worth mentioning that dynamic methods can automatically correct for FPN variations due to parameter and temperature changes.

Based on application area, cost, and performance requirements, many solutions were proposed for FFC implementation and calibration methods. As for the implementation of FFC in an FPGA, Vasiliev [21] describes an FPGA-based ISP for a VGA CMOS image sensor, including a column-based DSNU correction. For a basic FFC of the OmniVision OV5647 and Sony IMX219 sensor, Bowman et al. [22] proposed a simple apparatus to counter lens shading and establish color-correction coefficients.

To correct lens shading and sensor nonuniformity, many documented solutions propose static, single-reference solutions. This is suitable for applications such as microscopy (Zhaoning [23]), where at least temperature is expected to be relatively stable. The approach is also viable for IR FPAs, used by many consumer grade (Teledyne-FLIR [24]) and aerospace (Hercules [25]) IR cameras, which perform NUC periodically during operation using a cold-plate mechanical shutter (Orżanowski [26]). However, closing the shutter during use for a short period to capture FPN reference images may not be acceptable for defense or real-time process control applications. Another class of FFC solutions compensate for temperature but disregards the dependency of the DSNU and PRNU on analog gain (Yao, [27]).

## 5. Results

This section first introduces the proposed FFC architecture, suitable for an embedded implementation on FPGAs or ASIC ISPs, then reviews temperature and analog gain dependence of DSNU and PRNU measurements on the Sony IMX265 and IMX273 global shutter machine vision sensors. Fixed-pattern noise suppression results are presented for different FFC configurations.

### 5.1. Flat-Field Correction

In the pixel stream $N_{x,y}$, as defined by (12), the signal is coupled with the DSNU term $d_{x,y}$ and PRNU term $r_{x,y}$. In order to correct frames with the commonly used reference-based two-point calibration (TPC) method [26], the additive DSNU, and the multiplicative PRNU need to be removed by performing the following correction:

(19) $$O_{x,y}\left(\bar{p}\right)=G_{g}\left[N_{x.y}\left(\bar{p}\right)-Z_{in}-G_{d}\left(\bar{p}\right)d_{x.y}\left(\bar{p}\right)\right]\left[g_{x.y}\left(C,T\right)r_{x.y}\left(\bar{p}\right)\right]+Z_{out},$$

where $Z_{in}=\langle D\rangle$ is the black level of the sensor input frame, $Z_{out}$ is the expected output black level, $G_{d}\left(\bar{p}\right)$ is a temperature, gain, and sensor $\left(\bar{p}\right)$-dependent coefficient, which may need to be re-evaluated every time parameters, such as temperature or analog gain, change. During DSNU measurement, and consequently during regular use, the input black level, $Z_{in}$, is typically set in the sensor to several times the expected standard deviation of the DSNU to avoid clipping the measured FPN. Coefficients $G_{g}$ and $Z_{out}$ perform a linear transformation of the output pixel range, mapping values to the expected output range, 8–16 bits per pixel data.

**Figure 7:**
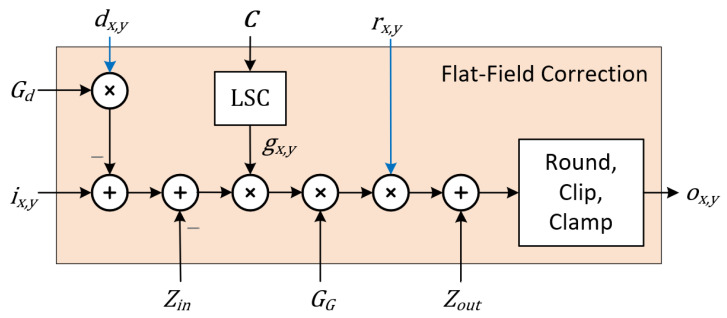
Flat-field correction module block diagram.

Figure 7 shows a block diagram of a single-channel FFC module. Inputs to the module are the sensor input pixels $i_{x,y}=\left[\alpha \rho N_{x,y}\right]$, where α is the analog gain applied, ρ is a charge-to-voltage coefficient, and [] denotes the quantization, clipping, and clamping of the sensor output. Internal to the FFC module is an optional, parametric lens shading correction (LSC) block, which can be configured with a 32 × 32 matrix C(T) of temperature-dependent coefficients.

**Figure 8:**
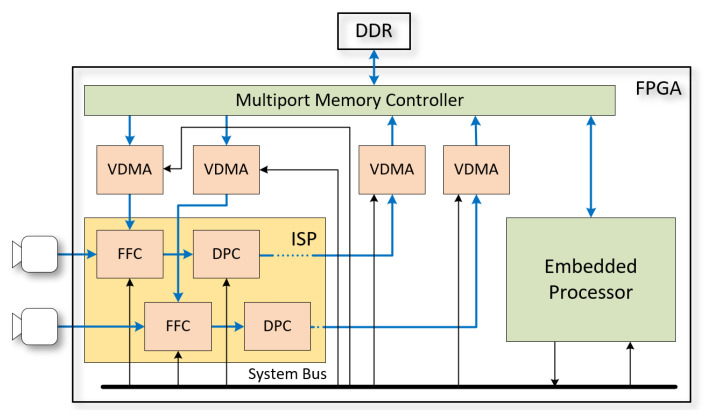
Uniformity correction of stereo cameras.

Parameters $G_{d}$, $G_{G}$, C(T), $Z_{in}$, and $Z_{out}$, which only change between frames, are provided by ISP FW. In the proposed FPGA implementation of an ISP for a stereo machine vision camera, the module maps efficiently to the DSP48 resources of novel Xilinx or Lattice FPGAs. Generic parameters controlling the instantiation of the DSNU, lens shading, and PRNU correction sections allow a balancing of FPGA logic resources with the performance requirement of the target application. For example, if a temperature-compensated lens shading correction is not a requirement, lens shading correction can be performed by the PRNU correction multiplier, if the frame buffer providing $r_{x,y}$ is initialized with the combined PRNU and lens shading correction image. All coefficients of the FFC hardware component need to be initialized before use, and coefficients $G_{d}\left(\bar{p}\right),C\left(T\right),d_{x,y}$, and $r_{x,y}$ may need to be regularly updated to match sensor operating parameters. The initialization and subsequent parameter updates are carried out by firmware (FW), which in the proposed implementation are executed by an embedded processor collocated in the same multiprocessor system on a chip (MPSoC) FPGA as the ISP HW (Figure 8). Besides configuring FFC parameters, FW also configures the sensor(s), which includes programming the black level to the same $Z_{in}$ value provided to the FFC module corresponding to the sensor.

Per-pixel DSNU and PRNU correction reference frames $d_{x,y}$ and $r_{x,y}$ are provided to the HW FFC modules from external memory (DDR). The video direct memory access (VDMA) modules in the system transfer video frames between the memory controller and other system components. The memory controller provides a shared access to external memory, by arbitrating and prioritizing requests. The VDMAs are also configured by the system processor, and cyclically write or read frames from predetermined memory address ranges. During initialization, one, or many DSNU and PRNU calibration images, pertinent to different temperature and analog gain settings, can be loaded to the DDR memory. During operation, FW programs exposure and analog gain settings into the image sensors for every frame (autoexposure) and periodically reads sensor temperatures. Based on temperature and gain settings, it may also reconfigure the VDMAs to select PRNU and DSNU images best matched to the operating conditions and update the parametric lens shading model coefficients (C) based on temperature.

The DDR memory bandwidth is a scarce resource, shared by the VDMA modules, the system processor, and other ISP modules and HW accelerators implemented in the MPSoC. As the high-speed components of the external memory interface subsystem are primary drivers of dissipation and power consumption, a secondary goal of an efficient FFC solution is to minimize the DDR memory bandwidth. Another design objective is to minimize manufacturing and calibration time. Capturing DSNU and PRNU images for large sets of gain–temperature combinations for each individual sensor can be prohibitively costly for mass manufacturing practical machine vision systems.

The subsequent analysis and summary focus on DSNU and PRNU reduction over the entire gain and temperature range served by the ISP, while simultaneously minimizing access to the DDR memory and the number of calibration images used.

### 5.2. DSNU Analysis

DSNU measurements without the lens holder and lens assembly were conducted with a cover over the sensor. DSNU measurements with the lens assembly attached were conducted with the lens cap covering the lens.

#### 5.2.1. DSNU and Exposure Time

In the first dataset DSNU images for the IMX265LLR-C and IMX273LLR-C sensors were captured at 0.5 ms and 2 ms exposure times, while holding the temperature and gain constant. Table 1 demonstrates almost perfect correlation between frames captured with different exposure times, with an almost identical standard deviation (SD). The SD is expressed in LSBs of 12-bit sensor data.

The dark current (thermal noise) was attenuated, but not fully canceled by averaging. Note that the lower correlation values pertained to parameter sets with a low SD, which amplified the relative effects of quantization and residual temporal noise. Likely these sensors already contained advanced silicon processes and features to reduce dark current and to compensate for biases. These findings were consistent with the findings of Changmiao [28]. Based on the results, for the rest of the analysis, the DSNU was treated as invariant with respect to the exposure time.

#### 5.2.2. Standard Deviation of Uncorrected DSNU

**Figure 9:**
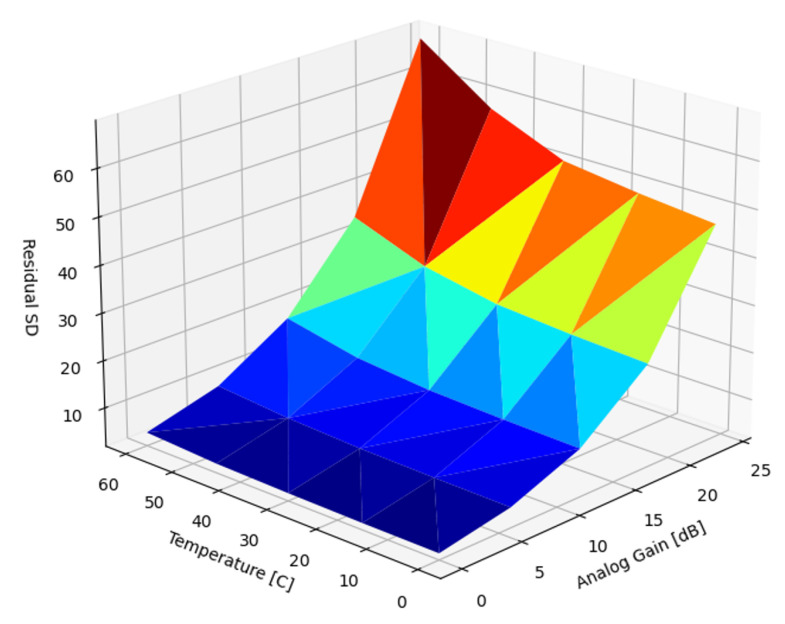
Standard deviation of uncorrected DSNU.

Figure 9 presents the SD of the uncorrected DSNU as a function of temperature and analog gain. Matching expectations and existing results, the magnitude of the DSNU scaled exponentially with both temperature and analog gain.

#### 5.2.3. Single-Point Correction

**Figure 10:**
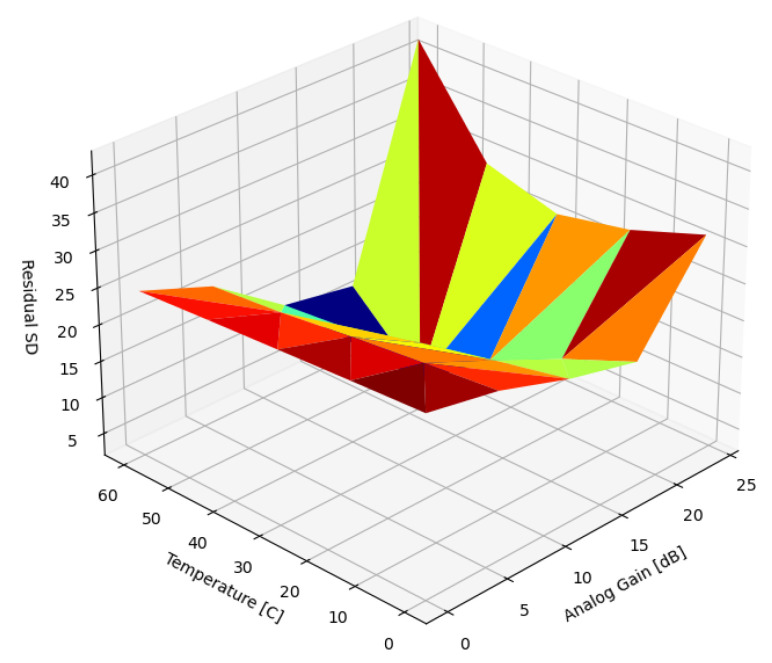
Residual SD of DSNU with a single, static image.

As a first approximation a single prior, ${\hat{d}}_{x,y}$, was used without adjusting the magnitude ($G_{d}$) to cancel the DSNU across the entire temperature and analog gain range. Figure 10 presents the SD of the residual DSNU when corrected with a static reference image captured at 45 C and 18.0 dB. At these parameter values, the SD of the DSNU was 28.64 (Table 2), about half of the worst-case SD.

As expected, due to the strong correlation between the DSNU across temperatures and gain ranges, the DSNU was almost perfectly canceled at parameter values close to reference image capture conditions. The nonzero residual noise was due to the fact that two sets of 2000 images were collected for each parameter setting, and one stack was corrected using the other as reference. This method also helped quantify leftover temporal noise in the data. The worst-case DSNU was reduced considerably, by 37%, but the *DSNU is significantly increased* in the range of the parameter space where the SD of the DSNU was lower than that of the reference image. Pearson’s correlation was chosen as a similarity metric between FPN captures due to its invariance to signal magnitude. Pearson’s correlation between a scaled reference frame and an actual dark input frame remained unaffected by scaling with ($G_{d}$).

**Figure 11:**
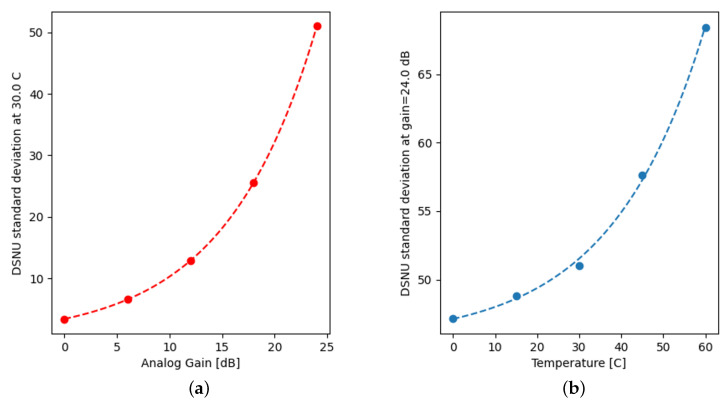
Standard deviation of DSNU at 30 °C (**a**) and at a gain of 24.0 dB (**b**).

Equation (16) establishes the theoretical background for the exponential relation with temperature, while the definition, $\alpha =20log_{10}A_{g}$ (dB) of analog gain consequently results in α, used by the programmable gain amplifiers in the sensor to scale exponentially with the $A_{g}$ factor as shown in Figure 11a.

By fitting exponentials along the axes (Figure 11) and modeling DSNU as a product of an FPN template, with the magnitude approximated with a separable surface yielded:

(20) $$d_{x,y}\left(T,\alpha \right)\approx {\hat{d}}_{x,y}D\left(T,\alpha \right)$$

(21) $$D\left(T,\alpha \right)=c_{0}\left(c_{1}e^{c_{2}\alpha }+c_{3}\right)\left(c_{4}e^{c_{5}T}+c_{6}\right)$$

where ${\hat{d}}_{x,y}$ is a reference DSNU capture, normalized to σ = 1.0. For optimal results ${\hat{d}}_{x,y}$ should be captured at a temperature and gain setting maximizing correlation with DSNU images captured for the rest of the parameter space.

For the IMX265, $c_{0,1,\ldots ,6}$ = {0.0196, 3.1605, 0.1156, 0.2679, 1.547, 0.0449, 45.5775}.

Considering the high correlation of FPN patterns for different parameters (T,α), FPN suppression could be significantly improved by scaling the reference DSNU image captured using the parametric approximation model of Equation (20).

**Figure 12:**
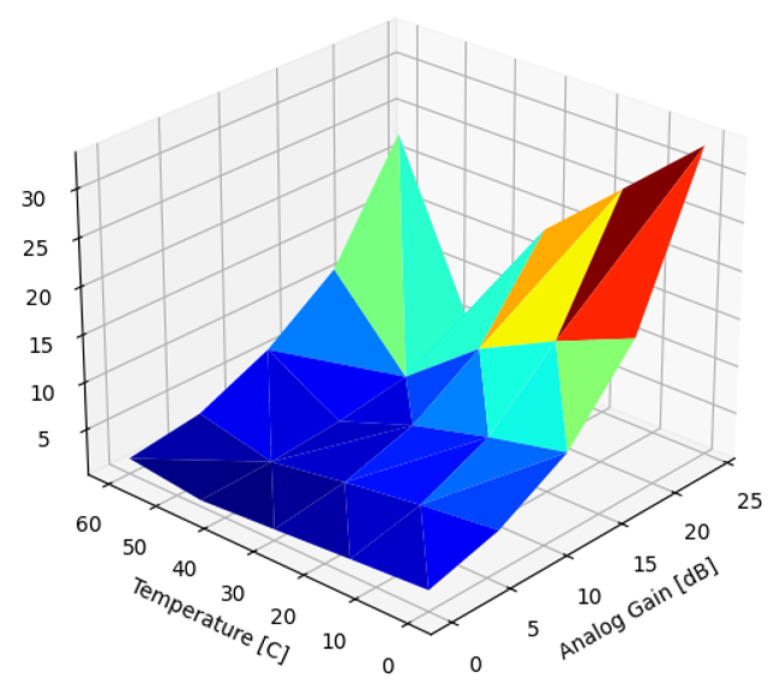
Standard deviation of DSNU corrected with a single, scaled image.

Figure 12 presents the standard deviation of the residual DSNU after correction with a single reference that was scaled by

(22) $$G_{d}\left(T,\alpha \right)=\frac{D(T,\alpha )}{\sigma ({\hat{d}}_{x,y})},$$

where D(T,α) is the approximation introduced in Equation (21), and $\sigma ({\hat{d}}_{x,y})$ is the SD of the reference frame. This method reduced the DSNU across the entire temperature and gain parameter space. The best DSNU reduction performance, 91.39% (21.3 dB), coincided with the temperature and gain identical to the reference frame parameters, with the highest Pearson correlation between frame and reference. The worst reduction performance, 25.1% (2.51 dB), was measured at the parameter combination with the least Pearson correlation with the reference frame. Establishing D(T,α) for each sensor instance may be prohibitively costly for mass manufacturing. While D(T,α) is fairly uniform for the same batch of sensors, a more accurate method is to read out the optically blanked pixels (OBP) around the active region of the sensor image frame, then calculate the standard deviation of the OBP region. OBPs are affected by temperature and gain settings identical to regular pixels and provide an accurate value for in situ assessment of SD magnitude. This method can be considered a digital postprocessing step after the analog correction proposed by Zhu [29]. Reading out the OBP presents a small (~1%) overhead during regular operation, which is a trade-off to be considered with the manufacturing overhead of establishing D(T,α).

#### 5.2.4. Multipoint Correction

Results from correction with a single reference frame have confirmed that the key to improved FPN reduction performance is to use reference frames better correlated with the FPN characteristic to the temperature and gain parameters of the image frames to be corrected. For this purpose, many IR thermal imaging sensors and staring cameras use multiple sets of FPN reference images and apply the one best suited to actual operating parameters.

#### 5.2.5. Linear Interpolation between Multiple Reference Images

A straightforward way to improve correlation is to calibrate at multiple gain and temperature settings, then interpolate the reference frame used by FFC HW based on current temperature and gain settings (T,α). Suppose n≥3 reference points, {$p_{1},p_{2},\ldots ,p_{n}$}, in parameter space $\bar{p}$ are selected for calibration, for which the corresponding DSNU reference images ${\hat{d}}_{i}\in \left\{{\hat{d}}_{1},{\hat{d}}_{2}\ldots {\hat{d}}_{n}\right\}$ have been captured. To find the estimated $\hat{d}$ pertinent to p={a,T}, we need to find $p_{A},\;p_{B}$ and $p_{C},$ the three closest neighbors to *p*, defining a triangle that preferably contains *p*. Operator $∥p-p_{i}∥=(c{\left(\alpha -\alpha_{i}\right)}^{2}+{\left(T-T_{i}\right)}^{2}$ can be used to rank order all candidate reference points by proximity, where α and *T* are the analog gain and temperature for *p*, $\alpha_{i}$ and $T_{i}$ are the analog gain and temperature for reference point $p_{i}$, and *c* is a constant scalar. Note that the perfect distance metric would be the inverse of the correlation between the DSNU of the particular frame and the DSNU of the reference frame; however, the DSNU of the current frame is unknown. Based on Table 2, the analog gain only scales DSNU; therefore, the DSNU along the gain axis is highly correlated, suggesting a low value for *c*. The resulting reference frame to be used for FFC is interpolated using

(23) $$\hat{d}=\sum_{i\in \{A,B,C\}}\lambda_{i}{\hat{d}}_{i},$$

where $\lambda_{i}$ are the barycentric coordinates of *p*, as defined by the triangle formed by $p_{A},p_{B}$ and $p_{C}$ in the analog gain and temperature parameter space $\bar{p}=\left\{a,T\right\}$. $\lambda_{i}$ can be found by solving

(24) $$P\bar{\lambda }={\bar{p}}^{\prime }$$

(25) $$\left[\begin{array}{ccc} 1 & 1 & 1\\ a_{A} & a_{B} & a_{C}\\ T_{A} & T_{B} & T_{C} \end{array}\right]\left[\begin{array}{c} \lambda_{A}\\ \lambda_{B}\\ \lambda_{C} \end{array}\right]=\left[\begin{array}{c} 1\\ a\\ T \end{array}\right]$$

for $\bar{\lambda }$, which yields

(26) $$\left[\begin{array}{c} \lambda_{A}\\ \lambda_{B}\\ \lambda_{C} \end{array}\right]=\left[\begin{array}{c} (\left(\lambda_{B}-\lambda_{C}\right)\left(T-T_{C}\right)+\left(\lambda -\lambda_{C}\right)\left(T_{C}-T_{B}\right))/D\\ (\left(\lambda_{C}-\lambda_{A}\right)\left(T-T_{C}\right)+\left(\lambda -\lambda_{C}\right)\left(T_{C}-T_{A}\right))/D\\ 1-\lambda_{A}-\lambda_{B} \end{array}\right]$$

(27) $$D=\left(\lambda_{C}-\lambda_{B}\right)\left(T_{A}-T_{C}\right)+\left(\lambda_{A}-\lambda_{C}\right)\left(T_{B}-T_{C}\right)$$

The magnitude of D can be thought of as the oriented area of a parallelogram defined by the $p\overset{AB}{\to }$ and $p\overset{AC}{\to }$ vectors. If $p_{A},p_{B}$ and $p_{C}$ are on a line, then D = 0. The larger |D| is, the more orthogonal $p\overset{AB}{\to }$ and $p\overset{AC}{\to }$ are, thus the better for interpolating $\bar{p}$. Another consideration besides having D≠0 for selecting three candidates from the rank-ordered list of reference candidates is to have positive barycentric coordinates $\left(\lambda_{i}>0\right),\forall i\in \left\{A,B,C\right\}$, which ensures $\hat{d}$ is not extrapolated in Equation (23).

#### 5.2.6. Single-Point Correction

The SD of the resulting blended reference $\hat{d}$ can be calculated during the evaluation of (23), or can be estimated using precomputed SDs of the constituent references:

(28) $$\sigma \left(\hat{d}\right)\approx \sqrt{\sum_{i\in \{A,B,C\}}\lambda_{i}\sigma^{2}({\hat{d}}_{i}}),$$

**Figure 13:**
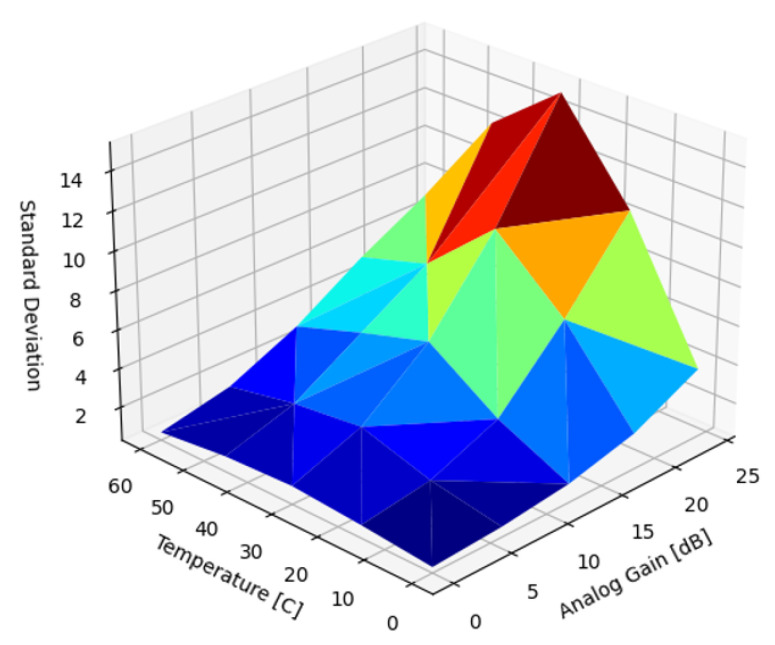
SD of DSNU corrected with linearly interpolated reference, using 4 reference captures.

Similar to the method introduced for a single reference, the estimated SD can be used to scale the interpolated reference frame $\hat{d}$ according to (22). To evaluate the linear interpolation method, four reference DSNU images were captured at the four corners of the parameter space, using both extremes of temperature and gain. This intuitive selection ensured that for any parameter combination *p* in parameter space $\bar{p}$, a set of three references could be selected such that *p* was inside the triangle defined by the references. Figure 13 shows that interpolating the reference image produced very good results, significantly reducing the worst-case DSNU.

#### 5.2.7. Optimizing DSNU Reference Selection

**Figure 14:**
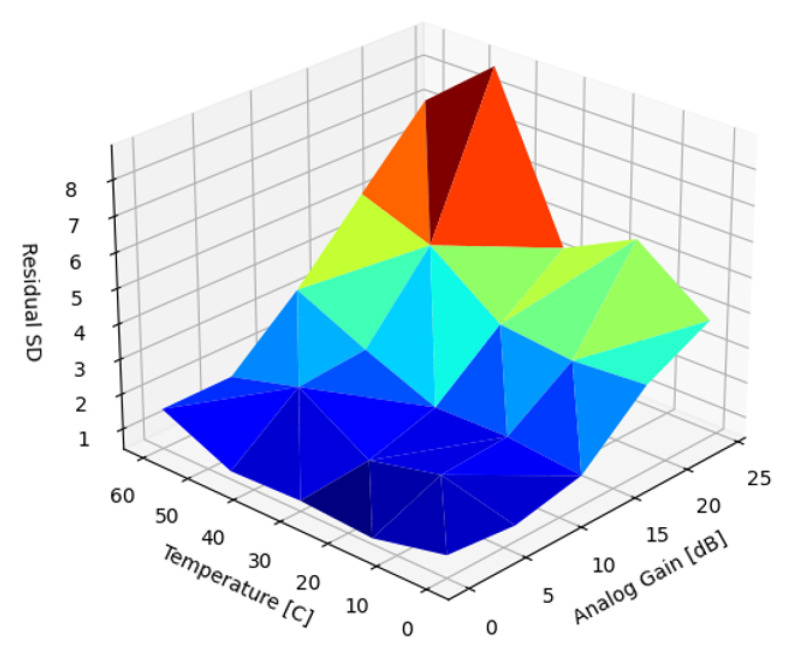
SD of DSNU corrected with linearly interpolated reference, using 5 reference captures.

In order to interpolate between references, at least three reference DSNU captures are needed. Intuitively, by each additional reference image captured, we can minimize the DSNU for the parameter combinations of the reference image, at the cost of additional calibration time, DDR memory allocation, and boot time. DSNU suppression quality can be also improved by optimizing the reference parameters for a given number of reference frames. Figure 14 presents the residual SD after correction with five reference images, captured at (0 °C, 0 dB), (60 °C, 0 dB), (0 °C, 24 dB), (30 °C, 24 dB), and (60 °C, 24 dB).

Table 3 lists the Pearson correlation values between the actual frames and the interpolated references. Besides improved DSNU suppression measurements, notice the improvement in the correlation with respect to the correlation values in Table 2.

Every time the analog gain or measured die temperature deltas exceed a predefined threshold, the embedded processor controlling the ISP needs to recompute the interpolated reference image $\hat{d}$. Selecting the three closest candidates around the current p={α,T} from a set of $\left\{p_{1},p_{2}\ldots p_{n}\right\}$ reference parameters is trivial. The majority of the FFC-related workload for the embedded ISP processor is to perform the actual interpolation on millions of pixels, which is dependent on the frame size, but invariant regarding the number of reference images, *n*. At startup, the embedded processor needs to load reference DSNU frames from the nonvolatile memory (NVME) to the system memory (DDR), which, depending on the NVME used may present a small penalty in terms of boot time, for each additional reference image. DDR memory or NVME’s size/cost is typically not a concern considering image sizes relative to current package capacities. In order to optimize the locations of reference captures $\left\{p_{1},p_{2}\ldots p_{n}\right\}$ in parameter space $\bar{p}=\left\{\alpha ,T\right\}$, we need to introduce the following quantities:

- Let $\psi_{\bar{\epsilon }}\left(\alpha^{\prime },T^{\prime }\right)$ denote the probability that during regular operation, the sensor temperature (*T*) and analog gain (α) are within a predefined range $|T-T^{\prime }|<\epsilon_{T}$ and $|\alpha -\alpha^{\prime }|<\epsilon_{a}$, such that
  (29) $$\sum_{\alpha_{min}}^{\alpha_{max}}\int_{T_{min}}^{T_{max}}\psi_{\bar{\epsilon }}\left(\alpha ,T\right)dT=1.0$$
  $\psi_{\bar{\epsilon }}$ is essentially the 2D probability density function based on discrete parameter α, which is a register setting, and continuous parameter T, derived from camera usage statistics.
- Let ω(α,T) denote the weight or relative importance of the user application, e.g., disparity mapping, associated with parameter combination (α,T). For high-gain scenarios, an increased temporal noise may reduce the importance of DSNU.

With these quantities, we can now select the optimum set of reference parameters, $\bar{p}$, defined by:

(30) $$\hat{p}=\arg\min_{p\in \bar{p}}\sum_{\alpha_{min}}^{\alpha_{max}}\int_{T_{min}}^{T_{max}}\sigma_{p}\left(\alpha ,T\right)\omega \left(\alpha ,T\right)\psi_{\bar{\epsilon }}\left(\alpha ,T\right)dT,$$

where $p=\{p_{1},p_{2}\ldots p_{n}\}$ is the set of (α,T) parameters at which reference images were captured. $\sigma_{p}\left(\alpha ,T\right)$ is the SD of the residual DSNU on images corrected with reference set *p*. The argmin operation can be implemented with the simulated annealing (SA) by Kirkpatrick [30], starting with *p* chosen from a constellation of parameters distributed along the edges of the parameter space. This offline operation may be time-consuming even on a powerful computer, but it only has to be performed once per reference set *p* during system design, assuming ω(a,T) and $\psi_{\bar{\epsilon }}\left(a,T\right)$ are stable.

#### 5.2.8. Correction with Logarithmic Interpolation

Since DSNU is an exponential function of both α and *T*, interpolating in logarithmic space intuitively may improve results:

(31) $${\hat{d}}^{\prime }=\exp\left(\sum_{i\in \{A,B,C\}}\lambda_{i}{\hat{d}}_{i}^{\prime }\right),$$

where ${\hat{d}}_{i}^{\prime }=\ln{\hat{d}}_{i}$ are the reference DSNU images stored in logarithmic format. Results unfortunately did not confirm this hypothesis, and with the same set of reference parameters, the resulting DSNU residuals were slightly higher than that of the linear interpolation.

### 5.3. PRNU Analysis

Noise patterns on the sensor output image depend on imaging conditions: illumination, exposure time, analog gain, temperature, and conducted, capacitive, and inductive electronic interference. The first set of experiments were designed to determine which imaging parameters affected the PRNU.

#### 5.3.1. PRNU and Exposure Time

FF images were captured for multiple IMX265LLR-C and IMX273LLR-C sensor instances at 0.5 ms and 2 ms exposure times, while holding the temperature and gain constant. Measurements were performed with the lens assembly present over the sensor. Table 4 demonstrates the almost perfect correlation between FF image stacks captured with different exposure times.

For the IMX265, the SD displayed only a slight dependence on exposure time and temperature, a 0.1% increase at 0 °C, and a 2.1% increase at 60 °C, while the exposure time increased fourfold from 0.5 ms to 2.0 ms. These increases were under the residual noise floor after averaging the temporal noise over 2000 images. The IMX273 did not display any measurable dependence on exposure time. Based on these results, for the rest of this analysis, PRNU was treated as invariant with respect to the exposure time.

In comparison with Table 1, the SD of the PRNU was up to two orders of magnitude larger than that of the DSNU, and initially seemed a lot less dependent on analog gain or temperature. A second set of measurements were performed with a focus on temperature and gain dependence for the IMX265LLR-C, capturing the PRNU at analog gain levels {0, 6, 12, 18, and 24 dB} levels, at {0, 15, 30, 45, and 60} degrees Celsius temperatures. For each sensor, gain, and temperature combination, two sets of measurement data were recorded, based on N = 2000 frame captures in each set. Both sets contained mean images, $\mu_{1}\left(T,\alpha \right)$ and $\mu_{2}\left(T,\alpha \right)$, which were generated by averaging the captured images, as well as standard deviation $\sigma_{1}\left(T,\alpha \right)$ and $\sigma_{2}\left(T,\alpha \right)$ images, by calculating the SD for each pixel across the stack of N images. The per-pixel SD allowed the estimation of the residual temporal noise present in the data.

#### 5.3.2. PRNU and Exposure Time

Before continuing with the analysis of the noise on image stacks captured with the FF illumination, it was important to characterize noise sources. At higher illumination levels, temporal noise is dominated by shot noise, the collective effect of the quantum nature of light. The actual number of photons captured during the exposure period follows a Poisson distribution. If the mean number of photons captured is ν, then the SD of shot noise is $\sqrt{\nu }$. The maximum number of photons converted to electrons is limited by the full well capacity of the sensor, $\nu_{max}$, as well as the saturation level of the ADCs following the PGAs. Thus, to get to an expected digital output level, such as a 70% white level, with higher analog gains, fewer photons need to be captured. When using analog gain α, the SD of the shot noise associated with the photon flux is reduced by $\sqrt{\alpha }$ at the photodiode, but this noise, superimposed on the signal, is then amplified by the PGA.

(32) $$\sigma \left(\alpha \right)=\alpha \sqrt{\frac{\nu }{\alpha }}=\sqrt{\alpha \nu },$$

**Figure 15:**
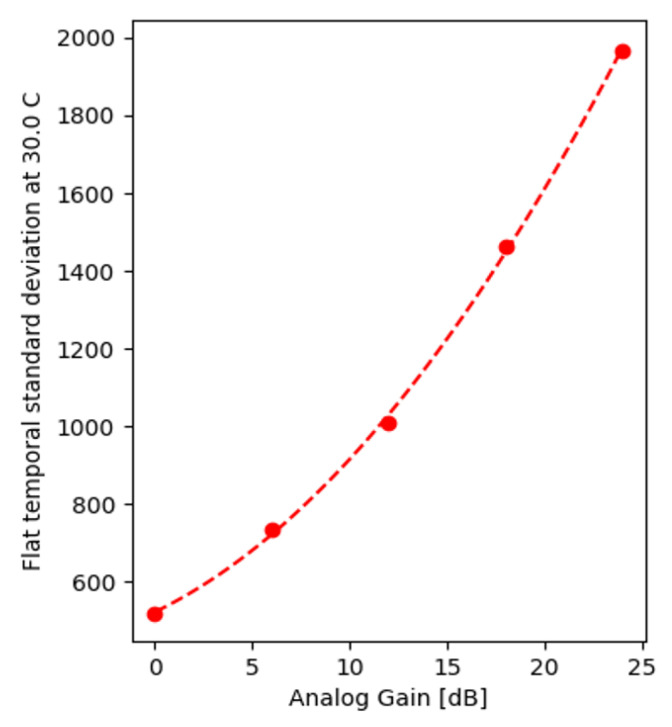
Shot noise as a function of analog gain.

Effectively, when comparing FF images captured with different analog gain settings resulting from similar output white levels, the SD is expected to scale with the square root of the gain applied. Figure 15, plotting the measured standard deviations $E[\sigma_{1}\left(T,\alpha \right)]$ on a lin–log scale, confirms this expectation. The 24.0 dB (maximum gain for the IMX265 and IMX273) applied a factor of 16 amplification, for which a 4× increase in shot noise was observed.

Averaging over *N* images reduces the SD of shot noise by a factor of $\sqrt{N}$. Based on SD measurements, the SD of temporal noise present in the image stack means $\mu_{1}\left(\alpha ,T\right)$ and $\mu_{2}\left(\alpha ,T\right)$ are expected to range from 11.2 LSBs (T=0 °C, α=0.0 dB) to 44.72 LSBs (T=60 °C, α=24.0 dB), due to averaging.

**Figure 16:**
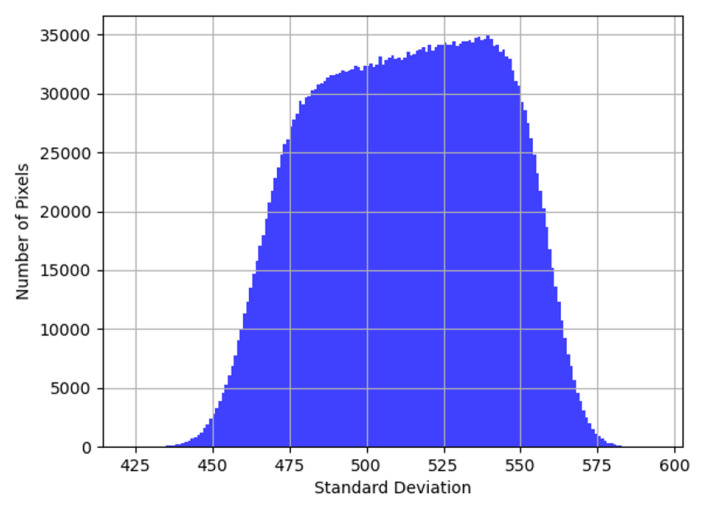
Distribution of per-pixel SD at T=30 °C, α=0.0 dB.

It is also worth noting that the distribution of per-pixel standard deviation $\sigma_{1}\left(\alpha ,T\right)$ is not necessarily Gaussian (Figure 16). Per the central limit theorem, the effects of multiple, uncorrelated noise sources with different means and SDs (such as shot noise, thermal noise, and electronic noise) superimposed on pixel outputs would present as a single Gaussian even if the distributions of the individual noise sources were not Gaussian. Different sensitivities translate to different photon counts and in turn, different SD distributions of shot-noise. For a monochrome sensor, with no lens assembly attached, Figure 16 is proof of a continuum of different sensitivities, which effectively is the definition of the PRNU present. To analyze noise variance on the pixel output, all temporal noise sources dependent on the illumination and gain, such as shot noise, were incorporated into $n_{s}\left(L,\alpha \right)$ and all other temporal noise sources, such as reset, electronic, thermal noise into $n_{T}\left(\alpha ,T\right)$. By factoring these into (11), with $N_{x,y}^{\prime }\left(\alpha ,T\right)$ as the output pixel value, assuming a uniform gray illumination (*L*), we obtained:

(33) $$N_{x,y}^{\prime }\left(\alpha ,T\right)=T_{int}R_{x,y}L\alpha +D_{x,y}\left(\alpha ,T\right)+n_{s}\left(L,\alpha \right)+n_{T}\left(\alpha ,T\right)$$

For FF measurements, exposure time $T_{int}$ was set such that the expected output value remained constant (70% of the white value, $N_{max}$) for the chosen illumination intensity *L* and analog gain α. Hence, $LT_{int}\alpha =0.7N_{max}$ was a constant factored into the PRNU, $r_{x,y}=T_{int}R_{x,y}L\alpha =0.7N_{max}R_{x,y}$. Since the DSNU for all analog gain and temperature combinations used in the PRNU analysis were recorded, captures could be corrected with the DSNU:

(34) $$n_{x,y}\left(\alpha ,T\right)=N_{x,y}^{\prime }\left(\alpha ,T\right)-D_{x,y}\left(\alpha ,T\right),$$

(35) $$n_{x,y}\left(\alpha ,T\right)=r_{x,y}+n_{s}\left(L,\alpha \right)+n_{T}\left(\alpha ,T\right),$$

Assuming statistical independence between the noise sources, the noise variance on the output could be expressed as:

(36) $$\sigma_{n}^{2}=\sigma_{r}^{2}+\sigma_{s}^{2}+\sigma_{T}^{2}=\sigma_{r}^{2}+\sigma_{t}^{2}$$

The first noise term, $\sigma_{r}$ was pertinent to the fixed pattern PRNU, which we aimed to minimize. The suppression of the temporal noise term $\sigma_{t}$ was beyond the scope of this paper.

**Figure 17:**
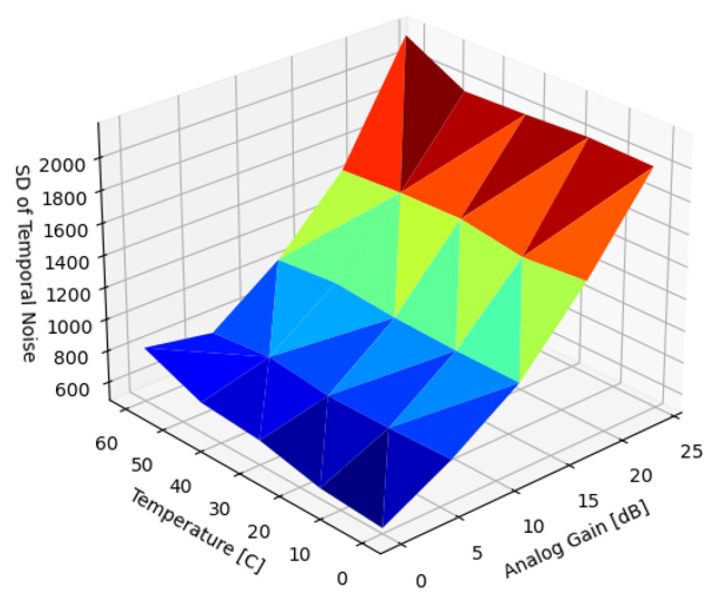
$\sigma_{t}\left(\alpha ,T\right)$ °C, α=0.0 dB.

Figure 17 illustrates the SD of the temporal noise as a function of the temperature and analog gain used. As expected, thermal noise $n_{T}\left(\alpha ,T\right)$ increased with temperature, and shot noise $n_{s}\left(L,\alpha \right)$ increased with analog gain α.

#### 5.3.3. Analysis of Flat-Field Image Stacks

The SD of the FF image stack $\left(\sigma_{n}\right)$ and temporal noise $\left(\sigma_{t}\right)$ were directly observable. When the two sets of mean images, $\mu_{1}\left(\alpha ,T\right)$ and $\mu_{2}\left(\alpha ,T\right)$, of the image stacks were combined:

(37) $$s\left(\alpha ,T\right)=\frac{\mu_{1}\left(\alpha ,T\right)+\mu_{2}\left(\alpha ,T\right)}{2}$$

The SD of the per-pixel temporal noise was suppressed by $\sqrt{2N}\approx 63.24$ and

(38) $$\sigma_{s}^{2}=\sigma_{r}^{2}+\frac{\sigma_{t}^{2}}{2N}$$

In the per-pixel *differences* of the two sets of mean images

(39) $$d\left(\alpha ,T\right)=\frac{\mu_{1}\left(\alpha ,T\right)-\mu_{2}\left(\alpha ,T\right)}{2}$$

the FPN term was eliminated, and the difference image capturing the residual temporal noise after averaging was:

(40) $$\sigma_{s}^{2}=\frac{\sigma_{t}^{2}}{2N}$$

#### 5.3.4. Standard Deviation of Uncorrected PRNU

From measurements of $\sigma_{s}\left(\alpha ,T\right)$ and $\sigma_{d}\left(\alpha ,T\right)$, the SD of the fixed-pattern component (PRNU) could be deduced:

(41) $$\sigma_{r}=\sqrt{\sigma_{s}^{2}-\sigma_{d}^{2}}$$

**Figure 18:**
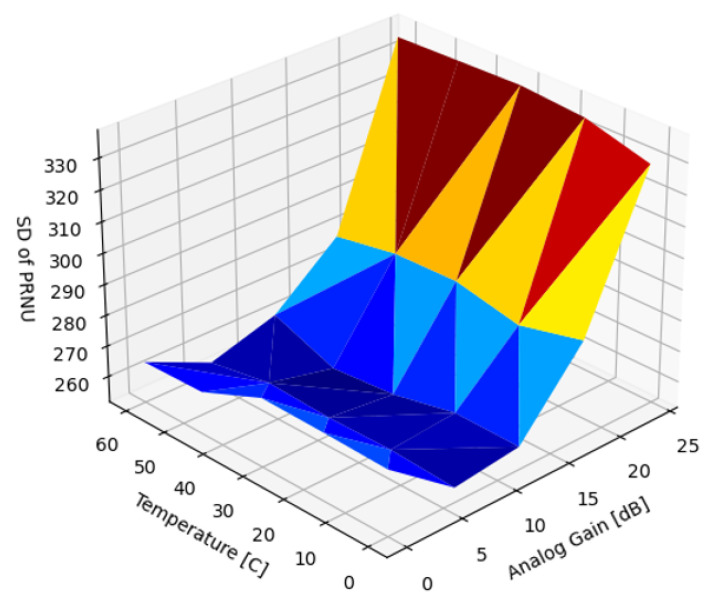
SD of uncorrected PRNU, $\sigma_{r}\left(\alpha ,T\right)$.

Figure 18 illustrates the dependence of the PRNU on the analog gain and temperature. It is also worth noting that the nonuniformity was only visible, though subtle, at very low gains where shot noise was at minimum. Above 0.5 dB gain, the FPN on the video was imperceptible as it was deeply buried in temporal noise.

#### 5.3.5. Single-Point Correction

**Figure 19:**
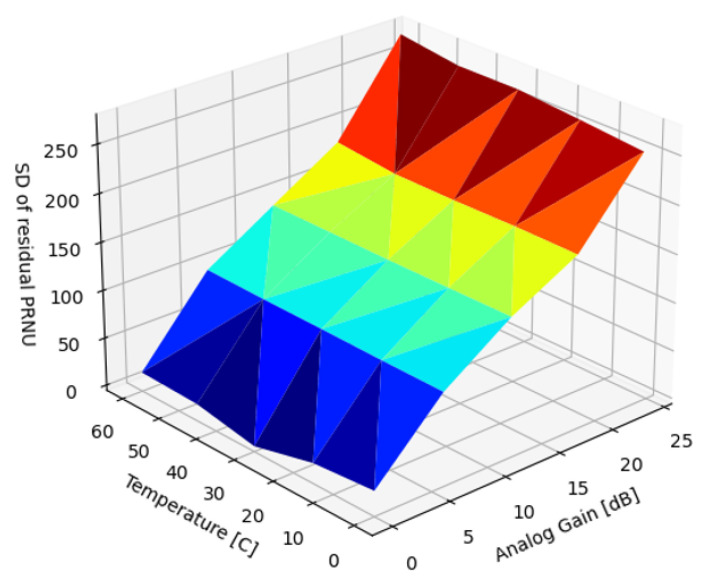
Residual SD of PRNU, single-reference correction.

Selecting a single correction image (Figure 19) is the simplest way to calibrate nonuniformity and has demonstrated good results (Yao, [27]). For suppression of the visible PRNU artifacts, selecting a calibration frame in the middle of the temperature range and at analog gain α=0.0 dB removed all visible artifacts.

**Figure 20:**
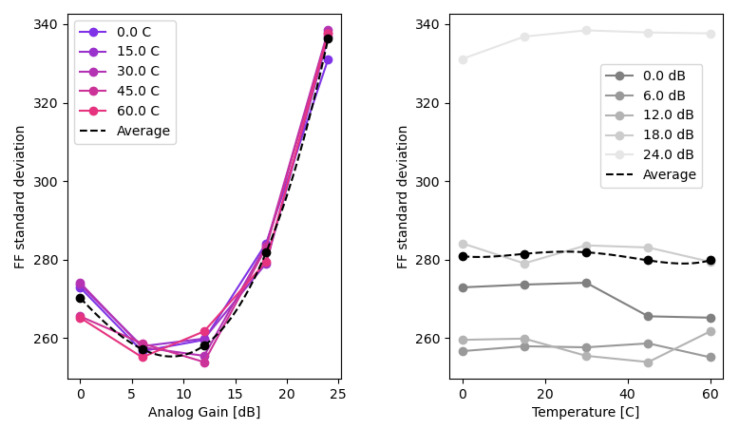
Projections and averages of $\sigma_{r}\left(\alpha ,T\right)$.

The analysis of the PRNU recorded at different temperatures and analog gains revealed a very high correlation across the entire temperature range. This augmented the findings of Figure 20, suggesting that the PRNU was stable across the operating temperature range.

#### 5.3.6. Multipoint Correction

**Figure 21:**
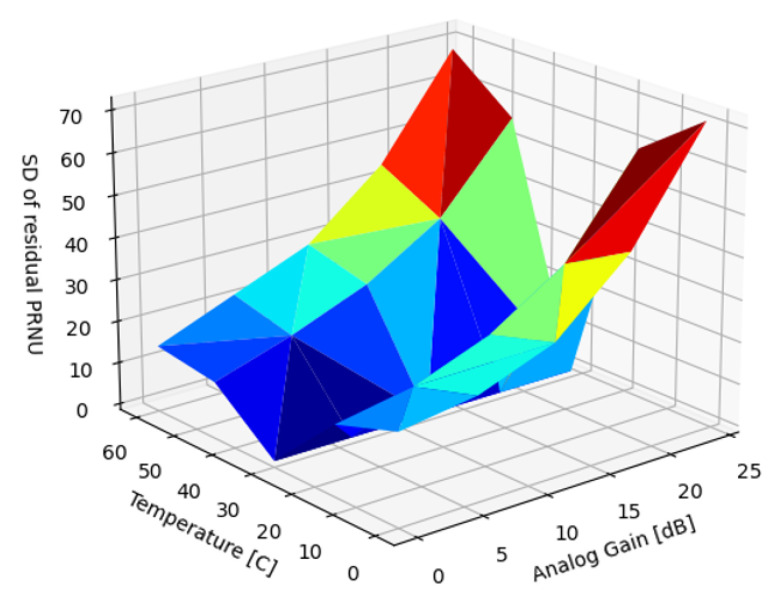
Residual SD of PRNU, multi-reference correction.

The results suggested that using multiple reference frames along the gain axis in the middle of the temperature range could minimize the SD over the entire parameter range (Figure 21).

Even though a multipoint correction can reduce the worst-case PRNU by a factor of four, a practical implementation of this method requires capturing multiple PRNU reference images in a temperature-controlled environment. The large number of images to capture for each image stack and reference image may be prohibitively expensive in a production environment.

## 6. Discussion

Based on the initial design objectives (FPN suppression performance, design silicon footprint, DDR memory bandwidth, and calibration complexity), and the insights gained from the analysis of factors affecting the DSNU and PRNU performance, the following solutions are recommended for different performance tiers:

1. The most egregious nonuniformity problem is uncorrected lens shading. For consumer products with inexpensive CMOS sensors and optics, such as webcams, a minimal ISP solution can use population images, captured once per manufactured batch, for lens shading correction, and no correction for DSNU or PRNU. Objectionable to human observers, and detrimental to machine vision and processing algorithms, lens shading can be compensated using just the parametric LSC module in the proposed FFC solution. This performance tier does not require an external frame buffer, VDMAs, or FW initialization of the correction buffers ($d_{x,y}$ and $r_{x,y}$).
2. For video applications where visible FPN is not acceptable, such as cell phones and DSLR cameras, the PRNU and DSNU has to be suppressed. This performance tier requires an external frame buffer, and VDMAs around the ISP block to provide $d_{x,y}$ and $r_{x,y}$. If fixed -focus optics are used, and the temperature compensation of the lens is not a requirement, LSC can be performed by convolving the intensity correction with PRNU correction in $r_{x,y}$. The results of Section 5.2.3 demonstrated that using a single, static image did not correct the DSNU sufficiently. As temperature and sensor gain change, this method may introduce more noise than originally present in the sensor image.
3. The top performance tier is suitable for high-end machine vision cameras, studio equipment, or computational photography where motion-compensated image stacks are registered to suppress temporal noise. For these demanding applications gain- and temperature-compensated DSNU, PRNU, and LSC are all utilized. Parametric LSC is suggested with module-specific, temperature-compensated lens shading parameters accounting for zoom and focus settings. For this tier, FW needs to either calculate D(T,α) or gather image statistics from the OBP region of the sensor and calculate σ(T,α) (Section 5.2). Moreover, FW may dynamically adjust the frame buffer contents to interpolate between DSNU and PRNU frames stored in DDR memory. As demonstrated in Section 5.2.3, DSNU correction can be significantly improved by using the global DNSU amplifier ($G_{d}$) feature of the FFC. PRNU suppression can be improved by using gain-dependent calibration images ($r_{x,y}$). For this performance tier, at initialization, multiple $r_{x,y}$ images need to be deposited into DDR memory by FW. During use, FW also needs to read out sensor temperature T, and based on the current analog gain setting α, update $G_{d}\left(\alpha ,T\right)$ and reprogram the VDMA read controller to point to the $r_{x,y}\left(\alpha \right)$ best matched to operating conditions.

## 7. Future Work

More work is necessary to study the stability of DSNU and PRNU images over time. It is well understood that the aging of image sensors due to high temperature and exposure to cosmic rays may introduce defective pixels during use. Very likely the same processes affect DSNU and PRNU, for which nonuniformity correction images need to be recalibrated periodically. Removing and reattaching the lens assembly to record PRNU images without LSC is problematic and may require access to a clean room and AA equipment, as the removal of sealing compounds may damage or contaminate the sensor assembly. Therefore, for the long-term use of sensor modules, DSNU and PRNU calibration with the lens assembly attached is much preferred.

## 8. Summary

In the introduction, we reviewed sources of nonuniformity in imaging systems: DSNU, PRNU and lens shading. In the subsequent Results section, we analyzed the dependency of the DSNU and PRNU of two modern, global shutter machine vision sensors on exposure time, die temperature, and analog gain (Figure 9 and Figure 18). In Section 5.1, we also provided an FFC architecture for ISPs, optimized for FPGA or ASIC implementation supporting different FPN suppression performance and resource use trade-offs. Based on different use case scenarios, the proposed FFC and ISP architecture could be configured for different performance tiers. For all performance tiers, the proposed architecture introduced minimal latency as images were read out from attached image sensors. The noise suppression performance of four different algorithms were quantified to suppress DSNU (Section 5.2), along with the analysis of the embedded software and calibration complexity of the different approaches. We provided methods (Section 5.2.7) for optimizing the capture parameters of reference captures. The performance of PRNU suppression with single and multiple reference captures were analyzed in Section 5.3.

## Figures and Tables

**Table 1:** DSNU standard deviations and Pearson correlations.

Sensor Type	Temperature (°C)	Analog Gain (dB)	Std. Dev. $T_{\mathrm{int}}=0.5$ ms	Std. Dev. $T_{\mathrm{int}}=2.0$ ms	Pearson Correlation
IMX265	10	2.0	1.423	1.416	0.974
IMX265	10	24.0	26.164	26.047	0.997
IMX265	50	2.0	3.275	4.982	0.983
IMX265	50	24.0	60.294	60.428	0.997
IMX273	10	2.0	4.430	4.416	0.988
IMX273	10	24.0	52.864	52.880	0.995
IMX273	50	2.0	4.799	5.376	0.962
IMX273	50	24.0	64.950	65.802	0.982

**Table 2:** SD and Pearson correlation for DSNU correction with a single static reference.

Temperature (°C)	Analog Gain (dB)	$T_{\mathrm{int}}$ (ms)	Original Std. Dev.	Residual Std. Dev.	Pearson Correlation
0	0	0.53	3.16	26.44	0.726
0	24	0.02	47.17	31.43	0.761
15	0	0.51	3.23	25.99	0.840
15	24	0.02	48.82	27.94	0.867
30	0	0.49	3.43	25.49	0.929
30	24	0.01	50.99	26.01	0.939
45	0	0.46	3.76	24.98	0.977
45	6	0.23	7.34	21.39	0.991
45	12	0.10	14.44	14.36	0.995
45	18	0.05	28.64	2.80	0.995
45	24	0.01	57.63	29.28	0.995
60	0	0.45	4.64	24.44	0.920
60	6	0.21	8.69	20.75	0.948
60	12	0.10	17.71	13.62	0.939
60	18	0.04	34.43	12.23	0.941
60	24	0.01	68.42	42.62	0.940

**Table 3:** Residual SD, Pearson correlation, and DSNU reduction using interpolation and 5 references.

Temperature (°C)	Analog Gain (dB)	Frame SD	Residual SD	Pearson Correlation	DSNU Reduction (%)	DSNU Reduction (dB)
0	12	11.90	1.28	0.994	89.3	19.39
0	24	47.17	3.82	0.997	91.9	21.84
0	18	23.61	2.89	0.993	87.8	18.24
0	6	6.05	0.87	0.990	85.7	16.88
0	0	3.16	1.06	0.944	66.6	9.52
15	24	48.82	5.33	0.994	89.1	19.24
15	18	24.40	2.73	0.994	88.8	19.01
⋮	⋮	⋮	⋮	⋮	⋮	⋮
45	12	14.44	2.28	0.987	84.2	16.02
45	6	7.34	2.12	0.958	71.1	10.80
45	0	3.76	0.65	0.985	82.8	15.30
60	24	68.42	7.10	0.995	89.6	19.69
60	18	34.43	5.17	0.989	85.0	16.47
60	12	17.71	3.21	0.984	81.9	14.84
60	6	8.69	1.56	0.984	82.0	14.90
60	0	4.64	1.55	0.944	66.6	9.52

**Table 4:** PRNU standard deviations and Pearson correlations.

Sensor Type	Temperature (°C)	Analog Gain (dB)	Std. Dev. $T_{\mathrm{int}}=0.5$ ms	Std. Dev. $T_{\mathrm{int}}=2.0$ ms	Pearson Correlation
IMX265	0	2.0	175.69	176.00	0.999141
IMX265	20	2.0	174.47	176.33	0.999050
IMX265	60	2.0	171.03	174.16	0.998582
IMX273	35	0.2	182.69	183.31	0.996231
IMX273	35	12.0	180.74	183.32	0.995280
IMX273	60	0.2	177.80	181.75	0.999082
IMX273	60	12.0	154.77	155.31	0.996216

## Data Availability

Data underlying the results presented in this paper are not publicly available at this time but may be obtained from the author upon reasonable request.

## Abbreviations

The following abbreviations are used in this manuscript:

ADC	Analog-to-digital converter
ASIC	Application-specific integrated circuit
CDS	Correlated double sampling
CMOS	Complementary metal–oxide semiconductor
CNN	Convolutional neural network
DDS	Differential delta sampling
DDR	Double data rate random-access memory
DSNU	Dark signal nonuniformity
FFC	Flat-field correction
FPA	Focal plane array
FPGA	Field-programmable gate array
FPN	Fixed-pattern noise
FW	Firmware
ISP	Image signal processor
IR	Infrared
LSC	Lens shading correction
LED	Light-emitting diode
MPSoC	Multiprocessor system on a chip
PGA	Programmable gain amplifier
PRNU	Photoresponse nonuniformity
RST	Reset
SD	Standard deviation
SEL	Select
SH	Sample and hold
SoC	System on a chip
TEC	Thermoelectric cooler
TX	Transmit
VDMA	Video direct memory access

## References

[1] Mooney J.M., Sheppard F.D., Ewing W.S., Ewing J.E., Silverman J. (1989). Responsivity Nonuniformity Limited Performance of Infrared Staring Cameras. Opt. Eng..

[2] Perry D.L., Dereniak E.L. (1993). Linear theory of nonuniformity correction in infrared staring sensors. Opt. Eng..

[3] Schulz M., Caldwell L. (1995). Nonuniformity correction and correctability of infrared focal plane arrays. Infrared Phys. Technol..

[4] Nakamura J. (2006). Dark Current. Image Sensors and Signal Processing for Digital Still Cameras.

[5] Seo M.W., Yasutomi K., Kagawa K., Kawahito S. (2015). A Low Noise CMOS Image Sensor with Pixel Optimization and Noise Robust Column-parallel Readout Circuits for Low-light Levels. ITE Trans. Media Technol. Appl..

[6] Kim M.K., Hong S.K., Kwon O.K. (2016). A Fast Multiple Sampling Method for Low-Noise CMOS Image Sensors with Column-Parallel 12-bit SAR ADCs. Sensors.

[7] Takayanagi I. (2006). Fixed Pattern Noise Suppression. Image Sensors and Signal Processing for Digital Still Cameras.

[8] Mei L., Zhang L., Kong Z., Li H. (2018). Noise modeling, evaluation and reduction for the atmospheric lidar technique employing an image sensor. Opt. Commun..

[9] Teledyne (2019). EV76C661 1.3 Mpixel CMOS Image Sensor. https://imaging.teledyne-e2v.com/content/uploads/2019/02/DSC_EV76C661.pdf.

[10] EMVA (2016). European Machine Vision Association (EMVA) Standard 1288, Standard for Characterization of Image Sensors and Cameras. https://www.emva.org/wp-content/uploads/EMVA1288-3.1a.pdf.

[11] Scharstein D., Szeliski R. High-accuracy stereo depth maps using structured light. Proceedings of the 2003 IEEE Computer Society Conference on Computer Vision and Pattern Recognition.

[12] Liu L., Zhang T. (2016). Optics Temperature-Dependent Nonuniformity Correction Via *ℓ*_0_-Regularized Prior for Airborne Infrared Imaging Systems. IEEE Photonics J..

[13] Guan J., Lai R., Xiong A., Liu Z., Gu L. (2020). Fixed pattern noise reduction for infrared images based on cascade residual attention CNN. Neurocomputing.

[14] Karaküçük A., Dirik A.E. (2015). Adaptive photo-response non-uniformity noise removal against image source attribution. Digit. Investig..

[15] Burggraaff O., Schmidt N., Zamorano J., Pauly K., Pascual S., Tapia C., Spyrakos E., Snik F. (2019). Standardized spectral and radiometric calibration of consumer cameras. Opt. Express.

[16] Wang Y., Wan B., Fu G., Su Y. (2021). PRNU Estimation of Linear CMOS Image Sensors That Allows Nonuniform Illumination. IEEE Trans. Instrum. Meas..

[17] CAL3 (2016). Image Engineering “CAL3 V2 Data Sheet”. http://www.image-engineering.de/content/products/equipment/illumination_devices/cal3/downloads/CAL3_data_sheet.pdf.

[18] Seibert J.A., Boone J.M., Lindfors K.K. (1998). Flat-field correction technique for digital detectors. Proceedings of Medical Imaging 1998: Physics of Medical Imaging.

[19] Snyder D.L., Angelisanti D.L., Smith W.H., Dai G.M., Idell P.S., Schulz T.J. (1996). Correction for nonuniform flat-field response in focal plane arrays. Proceedings of SPIE 2827, Digital Image Recovery and Synthesis III.

[20] Ratliff B.M., Hayat M.M., Tyo J.S. (2003). Radiometrically accurate scene-based nonuniformity correction for array sensors. J. Opt. Soc. Am. A.

[21] Vasiliev V., Inochkin F., Kruglov S., Bronshtein I. Real-time image processing inside a miniature camera using small-package FPGA. Proceedings of the International Symposium on Consumer Technologies.

[22] Bowman R.W., Vodenicharski B., Collins J.T., Stirling J. (2020). Flat-Field and Colour Correction for the Raspberry Pi Camera Module. J. Open Hardw..

[23] Zhang Z., Wang Y., Piestun R., Huang Z.-L. (2021). Characterizing and correcting camera noise in back-illuminated sCMOS cameras. Opt. Express.

[24] Teledyne-FLIR (2022). 160 × 120 High-Resolution Micro Thermal Camera: Lepton 3 & 3.5. https://flir.netx.net/file/asset/15529/original/attachment.

[25] Silicon and Devices (2016). Hercules 1280 × 1024, 15 µm Pitch, Digital InSb MWIR. https://www.scd.co.il/wp-content/uploads/2019/04/HERCULES_brochure_v3_PRINT.pdf.

[26] Orżanowski T. (2016). Nonuniformity correction algorithm with efficient pixel offset estimation for infrared focal plane arrays. SpringerPlus.

[27] Yao P., Tu B., Xu S., Yu X., Xu Z., Luo D., Hong J. (2021). Non-uniformity calibration method of space-borne area CCD for directional polarimetric camera. Opt. Express.

[28] Hu C., Bai Y., Tang P. (2016). Denoising Algorithm for the Pixel-Response Non-uniformity Correction of a Scientific CMOS Under Low Light Conditions. Int. Arch. Photogramm. Remote Sens. Spat. Inf. Sci..

[29] Zhu Y., Niu Y., Lu W., Huang Z., Zhang Y., Chen Z. A 160 × 120 ROIC with Non-uniformity Calibration for Silicon Diode Uncooled IRFPA. Proceedings of the 2019 IEEE International Conference on Electron Devices and Solid-State Circuits (EDSSC).

[30] Kirkpatrick S., Gelatt C.D., Vecchi M.P. (1983). Optimization by Simulated Annealing. Science.

